# Workload and job satisfaction among Austrian pediatricians: gender and generational aspects

**DOI:** 10.1007/s00508-022-02050-x

**Published:** 2022-06-23

**Authors:** Daniela S. Kohlfürst, Thomas Zöggeler, Daniela Karall, Reinhold Kerbl

**Affiliations:** 1 Department of Paediatrics and Adolescent Medicine, Medical University of Graz, Graz, Austria; 2 Department of Paediatrics I, Innsbruck Medical University, Innsbruck, Austria; 3 Department of Paediatrics and Adolescent Medicine, General Hospital Hochsteiermark, Leoben, Austria

**Keywords:** Pediatric primary care, Working conditions, Survey, Workplace, Age

## Abstract

**Background:**

The aim of this study was to evaluate different factors that may contribute to workload and job satisfaction among Austrian pediatricians.

**Methods:**

We conducted an online survey with 16 questions and performed statistical analyses.

**Results:**

Of 375 participating pediatricians, 61% were female, 39% male, 61% clinicians, 21% panel doctors and 12% private doctors. Overall, job satisfaction was moderate (6 ± 2.4 on a positive scale of 0–10). Higher working hours (*p* = 0.014) and higher patient numbers (*p* = 0.000) were significantly associated with lower job satisfaction. Lowest satisfaction was described for administrative or other nonmedical work. Lack of time for patient consultation was also correlated with poor satisfaction. Pediatricians older than 65 years reported the highest job satisfaction whereas pediatricians between 55 and 65 years and younger than 36 years showed the lowest scores. Although male pediatricians worked significantly more often more than 40 h per week than females (75% vs. 53%, *p* = 0.000), female pediatricians were less satisfied about the proportion of administrative (*p* = 0.015) and other nonmedical work (*p* = 0.014).

**Conclusion:**

New working models considering less workload, particularly less nonmedical work and intensified collaboration between pediatric clinicians and practitioners are needed to allow more available time per patient, to increase job satisfaction and thus to raise attractivity for pediatric primary care.

## Background

Stress at work and excessive workload have been the most important occupational health challenges for decades. It is well known that healthcare professionals are exposed to significantly more stress than the general population [1]. The working environment of individual healthcare professionals and thus their individual workload varies widely. Feelings of job stress typically occur when working conditions are perceived to be too challenging to cope with [2]. Constant pathological stress during work leads to adverse consequences for workers’ health, such as mental health syndromes (e.g., burnout), psychosomatic complaints and physical illness [3, 4]. Studies showed that the rates of burnout and dissatisfaction in the medical profession are highest at the beginning and middle of the career [5]. As they advance in career, many medical doctors find better coping strategies and thus partially compensate for the high workload.

Most of these studies dealt with the general dissatisfaction of healthcare professionals. Only few studies dealt specifically with workload and job dissatisfaction and their correlating factors in pediatric medicine.

The working environment of healthcare professionals is constantly changing and the physician has to adapt to new circumstances. Since new technologies, new diagnostic options and new therapeutic concepts arise, pediatricians have to continuously “educate” themselves and adapt to these new circumstances in order to provide state of the art medicine for their young patients [6]. Long working days, psychological and physical stress at work and the pressure to provide top-quality medicine often lead to dissatisfaction and burnout. Studies confirmed the relatively high dissatisfaction among pediatricians and high rates of burnout, which again may lead to serious treatment errors [5, 7–10]. Finally, dissatisfaction with work environment may lead to complaints and resignation: Knowledge about these facts may subsequently prevent medical students from choosing this specialization (negative role model).

Personal satisfaction is a prerequisite to successfully practice the medical profession. Therefore, in recent years adequate work-life balance has become increasingly important [11]. This study aims to analyze the workload of pediatricians in Austria. In particular, we wanted to elaborate gender and generational differences. The main motivation for this study was the fact that Austria, like other countries, faces the problem to sustain pediatric primary care [12].

## Methods

An online survey was created using SurveyMonkey® (Momentive Inc., San Mateo, CA, USA) and an email invitation was sent to all members of the Austrian Society of Pediatrics and Adolescent Medicine in February 2020.

The survey comprised 3 personal questions including age, gender and workplace, 2 questions related to workload (average working time per week and average number of patients per week), 1 question about the collaboration between pediatric clinicians and practitioners and 10 questions related to job satisfaction. Questions about job satisfaction were answered on a scale of 0 (not at all satisfied) to 10 (very satisfied) and captured overall job satisfaction and several aspects that potentially contribute to job satisfaction: satisfaction with personal work-life balance, time for patient consultation, the proportion of administrative work and other nonmedical work, income and economic pressure, further and continuing education, working alone and the possibility to exchange knowledge and opinions with colleagues.

A total of 375 pediatricians (22% return rate) participated in this survey.

We used the χ^2^-test to compare frequency distributions between two categorical variables. Differences between groups were tested with Student’s *t*-test or analysis of variance (ANOVA). To assess linear correlations between two variables Spearman’s correlation coefficient was used. Statistical analyses and creation of graphs were performed with IBM SPSS Statistics Version 26. A *p*-value < 0.05 was considered statistically significant.

The study was performed in accordance with the Declaration of Helsinki and was approved by the Ethics Committee of the Austrian Society of Pediatrics. Informed consent was obtained from all participants.

## Results

Characteristics of all 375 responding pediatricians are shown in Table 1. The majority of participants were female (61%) and working in hospitals (61%). Significantly more females (77% and 69%) were represented within the young age groups (25–35 years and 36–45 years, respectively), while in the older age group (> 65 years) more males (78%) were represented. Most doctors (62%) reported more than 40 working hours per week. Males worked significantly more often above 40 h per week than females (74.8% vs. 53.3%, *p* = 0.000). Pediatricians older than 65 years worked significantly less often above 40 h per week than all other age groups (*p* = 0.000).

**Table 1:** Characteristics of 375 participating pediatricians

	Participants	Female^a^	Male^a^
	*n* (%)	*n* (%)	*n* (%)
*Age groups*			
25–35 years	66 (17.6)	51 (22.5)	15 (10.2)
36–45 years	98 (26.1)	68 (30.0)	30 (20.4)
46–55 years	97 (25.9)	52 (22.9)	44 (30.0)
56–65 years	96 (25.6)	52 (22.9)	44 (30.0)
65+ years	18 (4.8)	4 (1.8)	14 (9.5)
*Workplace*^a^			
Clinicians	228 (61.0)	153 (67.4)	75 (51.4)
Panel doctors	80 (21.4)	34 (14.9)	46 (31.5)
Private doctors	45 (12.0)	26 (11.5)	19 (13.0)
Other ^b^	21 (5.6)	14 (6.2)	6 (4.1)
*Average working hours per week*			
< 10 h	3 (0.8)	2 (0.9)	1 (0.7)
11–20 h	10 (2.7)	6 (2.6)	4 (2.7)
21–30 h	41 (10.9)	33 (14.5)	8 (5.4)
31–40 h	89 (23.7)	65 (28.6)	24 (16.3)
41–50 h	153 (40.8)	86 (37.9)	66 (44.9)
> 50 h	79 (21.1)	35 (15.4)	44 (29.9)

^a^Data on gender and workplace were available for 374/375 participants

^b^Other included combination of workplaces (clinic and private or panel praxis), school doctors, management positions, parental leave and retirement.

**Fig. 1:**
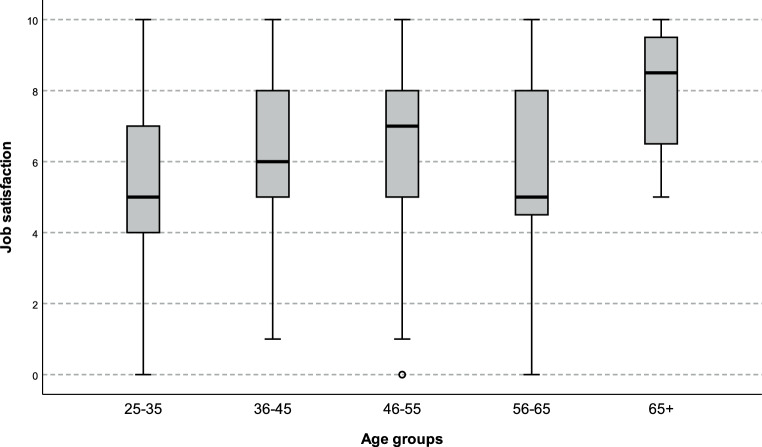
Job satisfaction in different age groups (years)

Pediatricans in hospitals worked significantly more often above 40 h per week than pediatricians in panel practices and private practices (*p* = 0.000). Pediatricians in panel practices worked more than pediatricians in private practices (*p* = 0.002). Furthermore, in panel practices more patients were seen per day compared to private practices (*p* = 0.000) or hospitals (*p* = 0.000). Overall, job satisfaction was moderate (6.0 ± 2.4 on a positive scale of 0–10). Pediatricians older than 65 years reported the highest job satisfaction, whereas pediatricians between 55–65 years and younger than 36 years showed the lowest scores (Fig. 1). Job satisfaction was significantly lower in panel practices (5.8 ± 0.3) and in hospitals (5.6 ± 0.2) compared to private practices (7.7 ± 0.3, *p* = 0.000).

Lowest satisfaction was described for administrative work (3.0 ± 2.3) and other nonmedical work (2.9 ± 2.5). Interestingly, this satisfaction was significantly lower in females than in males (*p* = 0.015 and *p* = 0.014, respectively, see Table 2).

**Table 2:** Rating of job satisfaction on a positive scale from 0–10

	Overall	Female	Male	
	Mean (SD)	Mean (SD)	Mean (SD)	*p*-value^a^
*Aspects of job satisfaction*				
Overall job satisfaction	6.0 (2.4)	5.8 (2.4)	6.3 (2.3)	0.077
Satisfaction with personal work-life balance	5.5 (2.3)	5.5 (2.2)	5.5 (2.4)	0.886
Satisfaction with time for patient consultation	4.2 (3.1)	4.3 (3.2)	4.1 (2.9)	0.622
Satisfaction with proportion of administrative work	3.0 (2.3)	2.7 (2.2)	3.3 (2.5)	**0.015**
Satisfaction with proportion of nonmedical work	2.9 (2.5)	2.7 (2.4)	3.3 (2.7)	**0.014**
Satisfaction with income	5.5 (2.8)	5.5 (2.7)	5.7 (2.9)	0.574
Satisfaction with economic pressure	4.6 (3.0)	4.4 (3.0)	4.8 (3.0)	0.291
Satisfaction with further and continuing education	6.6 (2.6)	6.5 (2.6)	6.7 (2.5)	0.473
Satisfaction with working alone	4.6 (2.9)	4.4 (2.8)	4.9 (3.0)	0.091
Satisfaction with exchange of knowledge with colleagues	5.2 (2.8)	5.3 (2.8)	5.1 (2.8)	0.472

Significant *p*-values are written in bold

^a^Student’s *t*-test

Higher working hours (*p* = 0.014) and higher patient numbers (*p* = 0.000) were significantly associated with lower job satisfaction. Similarly, other aspects of job satisfaction were associated with workload (Table 3). Higher working hours were significantly associated with dissatisfaction with personal work-life balance (*p* = 0.000), the lack of time for patient consultation (*p* = 0.000), the proportion of administrative work (*p* = 0.000) and the proportion of nonmedical work (*p* = 0.000). Higher patient numbers were significantly associated with dissatisfaction with personal work-life balance (*p* = 0.002), the lack of time for patient consultation (*p* = 0.000) and the possibility to exchange knowledge with colleagues (*p* = 0.000).

**Table 3:** Associations between workload and aspects of job satisfaction

	Average working time/week		Average number of patients/week	
	Spearman Rho (*n*)	*p*-value	Spearman Rho (*n*)	*p*-value
*Aspects of job satisfaction*				
Overall job satisfaction	−0.127 (372)	**0.014**	−0.226 (363)	**0.000**
Satisfaction with personal work-life balance	−0.283 (374)	**0.000**	−0.159 (365)	**0.002**
Satisfaction with time for patient consultation	−0.195 (370)	**0.000**	−0.431 (361)	**0.000**
Satisfaction with proportion of administrative work	−0.227 (372)	**0.000**	−0.093 (363)	0.087
Satisfaction with proportion of non-medical work	−0.212 (371)	**0.000**	−0.055 (362)	0.143
Satisfaction with income	0.067 (366)	0.201	−0.099 (357)	0.061
Satisfaction with economic pressure	−0.043 (353)	0.421	−0.042 (347)	0.435
Satisfaction with further and continuing education	−0.103 (371)	0.048	−0.052 (362)	0.320
Satisfaction with working alone	−0.084 (359)	0.111	−0.77 (353)	0.150
Satisfaction with exchange of knowledge with colleagues	−0.082 (371)	0.116	−0.275 (362)	**0.000**

Significant *p*-values are written in bold

## Discussion

We present the results of the first Austrian survey among pediatricians on job satisfaction with the main focus on gender and generational aspects. Thus far, there are only few studies on this topic worldwide and our results are mostly in line with data from these studies.

One limitation of our study is a response rate of 22%. Non-response might implicate potential bias. As the characteristics of our participants are very similar to characteristics of all members of the Austrian Society of Pediatrics and Adolescent Medicine (e.g., about 40% working in practices, < 20% below 35 years) we assume that this sample size is representative.

In our survey, most participants were female and at younger age, indicating that the field of pediatrics is very popular among young female doctors in Austria. This result is consistent with other studies [13–15]. Most pediatricians work more than 40 h a week, which is again in line with other studies showing that healthcare professionals spend more time at work than the average population [7, 16, 17]. The finding that males work significantly more often above 40 h per week than females may at least in part be explained by the higher number of women in part-time employment.

Overall, our survey showed an average level of 6 (on a scale of 0–10) for job satisfaction. Interestingly, pediatricians over 65 years of age showed the highest satisfaction, while early career pediatricians between the ages of 25 and 35 years reported the least satisfaction with work-life balance and overall job satisfaction. Also, this finding is in line with other studies and may be explained by the challenges at the beginning of the career. Recent studies from the USA have found that burnout rates and psychological stress are markedly higher amongst practicing physicians than in individuals in other careers, even when adjusting for working hours and other factors [7, 18, 19]. That means that the entry into a medical career is apparently particularly challenging. Life conditions may change dramatically and early career physicians have to take substantial responsibility within a very short time. The first night shifts may represent another burden. The first years of employment are therefore often characterized by disillusionment, self-doubt, disorientation and fear [17, 20–22]. In the further course, most pediatricians gain self-confidence and acquire further specialist knowledge, which helps to manage stress and to build resilience; however, in the middle years of the professional career other major challenges can have a negative impact on job satisfaction. Many doctors in private practice have to repay their loans for their practices. In private life, family planning and childcare may play a role, compatibility of family and work seems to be an essential cornerstone. The still relatively low job satisfaction of this age group may be explained by these factors.

Another interesting finding is the relatively low level (and a wide range) of job satisfaction in the age group 55–65 years. This may be interpreted as a kind of end of career dissatisfaction and the lack of positive future visions for many colleagues. In contrast, the clearly higher satisfaction level (and a narrow range) in the age group 65+years may be a hint that pediatricians voluntarily staying in their job over the age of 65 years are those who are primarily more satisfied and thus continue working. The finding that this age group worked significantly less often more than 40 working hours per week than all the other age groups might be a factor contributing to higher job satisfaction since we could show a clear overall association between less workload and higher job satisfaction. Interestingly, job satisfaction was higher in pediatricians older than 65 years regardless of workplace, however, sample size was low.

In all age groups, the lowest satisfaction was described for administrative work and other nonmedical work. It is a fact that the amount of doctors’ administrative work (such as documentation, writing letters, coordinating appointments) is steadily increasing, reducing the time left for medical tasks (such as interventions and special procedures) and direct patient contact.

Our survey did not show differences in overall job satisfaction between female and male pediatricians; however, females were significantly less satisfied with the proportion of administrative and nonmedical work than men. This finding suggests that female doctors are more engaged with administrative and nonmedical tasks, which could be explained by the fact that women are often employed in part-time positions after returning from maternal leave. The reason for this remains unclear and needs to be further addressed.

In our study, we found a clear association between high patient numbers and dissatisfaction with work-life balance and the possibility to exchange knowledge with colleagues. 90% of responders stated their wish for close cooperation with the regional children’s hospital(s) including repeated in-hospital training and possibly job rotation. Research on resilience indicates that such exchange of knowledge with colleagues and support within networks are important factors for job satisfaction and burnout prevention [23].

We could clearly see an association between higher workload in hospitals and panel practices and lower job satisfaction compared to private practices. This association needs to be addressed by policy and decision makers. The shortage of primary care pediatricians currently seen in several European countries enforces to develop new models of provision [24, 25]. These should consider the wishes and visions of the future generation of pediatricians.

## Conclusion

Our study suggests that less workload, particularly less administrative and other nonmedical work, more time for individual patients as well as intensified collaboration between pediatric clinicians and primary care pediatricians are important aspects to increase job satisfaction and thus to raise attractivity of paediatric primary care.
